# Consolidated lung on contrast-enhanced chest CT: the use of spectral-detector computed tomography parameters in differentiating atelectasis and pneumonia

**DOI:** 10.1016/j.heliyon.2021.e07066

**Published:** 2021-05-26

**Authors:** Philip Konietzke, Hauke H. Steentoft, Willi L. Wagner, Jonas Albers, Christian Dullin, Stephan Skornitzke, Wolfram Stiller, Tim F. Weber, Hans-Ulrich Kauczor, Mark O. Wielpütz

**Affiliations:** a Diagnostic and Interventional Radiology (DiR), Heidelberg University Hospital, Im Neuenheimer Feld 420, 69120 Heidelberg, Germany; b Translational Lung Research Center Heidelberg (TLRC), German Center for Lung Research (DZL), University of Heidelberg, Im Neuenheimer Feld 156, 69120 Heidelberg, Germany; c Department of Diagnostic and Interventional Radiology with Nuclear Medicine, Thoraxklinik at University of Heidelberg, Röntgenstraße 1, 69126 Heidelberg, Germany; d Institute for Diagnostic and Interventional Radiology, University Medical Center Göttingen, Robert-Koch-Straße 40, 37075 Göttingen, Germany

**Keywords:** Pneumonia, Atelectasis, Diagnosis, SDCT

## Abstract

**Objectives:**

To investigate the value of spectral-detector computed tomography (SDCT) parameters for the quantitative differentiation between atelectasis and pneumonia on contrast-enhanced chest CT.

**Material and methods:**

Sixty-three patients, 22 clinically diagnosed with pneumonia and 41 with atelectasis, underwent contrast-enhanced SDCT scans during the venous phase. CT numbers (Hounsfield Units [HU]) were measured on conventional reconstructions (CON_120kVp_) and the iodine concentration (C_iodine_, [mg/ml]), and effective atomic number (Z_eff_) on spectral reconstructions, using region-of-interest (ROI) analysis. Receiver operating characteristics (ROC) and contrast-to-noise ratios (CNRs) were calculated to assess each reconstruction's potential to differentiate between atelectasis and pneumonia.

**Results:**

On contrast-enhanced SDCT, the difference between atelectasis and pneumonia was significant on CON_120kVp_, C_iodine_, and Z_eff_ images (p < 0.001). On CON_120kVp_ images, a threshold of 81 HU achieved a sensitivity of 93 % and a specificity of 95 % for identifying pneumonia, while C_iodine_ and Z_eff_ images reached the same sensitivity but lower specificities of 85 % and 83 %. CON_120kVp_ images showed significantly higher CNRs between normal lung and atelectasis or pneumonia with 30.63 and 27.69 compared to C_iodine_ images with 3.54 and 1.27 and Z_eff_ images with 4.22 and 7.63 (p < 0.001). None of the parameters could differentiate atelectasis and pneumonia without contrast media.

**Conclusions:**

Contrast-enhanced SDCT can differentiate atelectasis and pneumonia based on the spectral parameters C_iodine_, and Z_eff._ However, they had no added value compared to CT number measurement on CON_120kVp_ images. Furthermore, contrast media is still needed for a differentiation based on quantitative SDCT parameters.

## Highlights

• Spectral CT parameters C_iodine_ and Z_eff_ can differentiate atelectasis from pneumonia on contrast-enhanced images.
• Spectral CT parameters C_iodine_ and Z_eff_ have no added value compared to CT number measurement on CON_120kVp_ images.
• CNRs between normal and consolidated lung were higher on conventional on spectral images.

## 1 Introduction

Pulmonary infections are responsible for significant morbidity and mortality worldwide, and clinical symptoms, laboratory tests, and imaging methods are used for diagnosis and therapy control [1, 2]. The ideal reference diagnosis for pneumonia is the detection of pathogenic agents in the lung parenchyma. However, invasive techniques like bronchoalveolar lavage or lung biopsy cannot be routinely performed for practical reasons.

Computed tomography (CT) can provide a regional and morphological description of lung pathologies and should be considered in patients with an unclear clinical condition or inadequate response to pneumonia therapy [2, 3]. Imaging signs of thoracic infection can be useful, sometimes suggesting a specific diagnosis and often narrowing the differential diagnosis. The consolidated lung is a common imaging sign of pulmonary infection, but it can also reflect atelectasis, a non-infectious lung pathology [4]. Radiological features like volume loss or a positive air bronchogram can help to differentiate pneumonia from atelectasis, but they remain qualitative, non-obligatory observations [4, 5]. In some clinical situations, the diagnosis of pneumonia is not unambiguous, and quantitative CT parameters would be desirable to facilitate a more confident diagnosis. The Hounsfield unit (HU), a relative quantitative measurement of x-ray density, is the most frequently used quantitative CT parameter, but unfortunately, the differences between atelectasis and pneumonia are usually not significant enough to allow a confident diagnosis on non-enhanced images. Here, contrast media administration can help since atelectasis shows a stronger contrast-enhancement than pneumonia [6]. In this context, Edwards *et al.* reported a threshold of 85 HU to diagnose pneumonia which reached a high 97 % sensitivity and 85 % specificity on contrast-enhanced CT pulmonary angiograms [6].

Spectral-detector computed tomography (SDCT) uses an X-ray tube and two different detector layers to selectively absorb different energies from the polychromatic X-ray spectrum [7, 8]. This technical approach allows for the simultaneous measurement of low and high-energy photons at the same spatial and angular location, facilitating dual-energy post-processing in the projection domain, different from other dual-energy techniques [9, 10, 11, 12]. The obtained spectral data set enables the retrospective analysis of the pixel-wise iodine concentration (C_iodine_) and the calculation of the effective atomic number (Z_eff_), reflecting the blood supply and the effective atomic number of inorganic materials. In the literature, SDCT parameters were already used to differentiate lung cancer from inflammatory masses and showed benefits when detecting pulmonary embolism and assessing pleural contrast uptake [13, 14, 15]. Due to the significantly different blood supply of atelectasis and pneumonia, we hypothesized that C_iodine_ and Z_eff_ images might have advantages over conventional images since they may offer additional information regarding perfusion properties. Therefore, we conducted this study to investigate if SDCT parameters C_iodine_ and Z_eff_ are beneficial compared to CT number quantification on conventional images for distinguishing atelectasis from pneumonia on contrast-enhanced chest CT.

## 2 Materials and methods

### 2.1 Patient cohort

This retrospective study was approved by the institutional ethics committee (S-781/2018), and informed consent for data processing was waived. Database research encompassing the years 08/2017 - 06/2020 identified 3167 patients who underwent venous phase contrast-enhanced or non-enhanced chest SDCT. CT acquisitions were serially evaluated for inclusion and exclusion criteria. The inclusion criteria were (1) radiologic feature of the consolidated lung, (2) consolidated lung >1 cm^2^ in size on four continuous slices, (3) age >18, and (4) absence of motion artifacts. The exclusion criteria were (1) tumor (>1 cm^2^), (2) radiologic features of atypical pneumonia, (3) metastatic lung disease, or (4) previous lung surgery.

**Figure 1:**
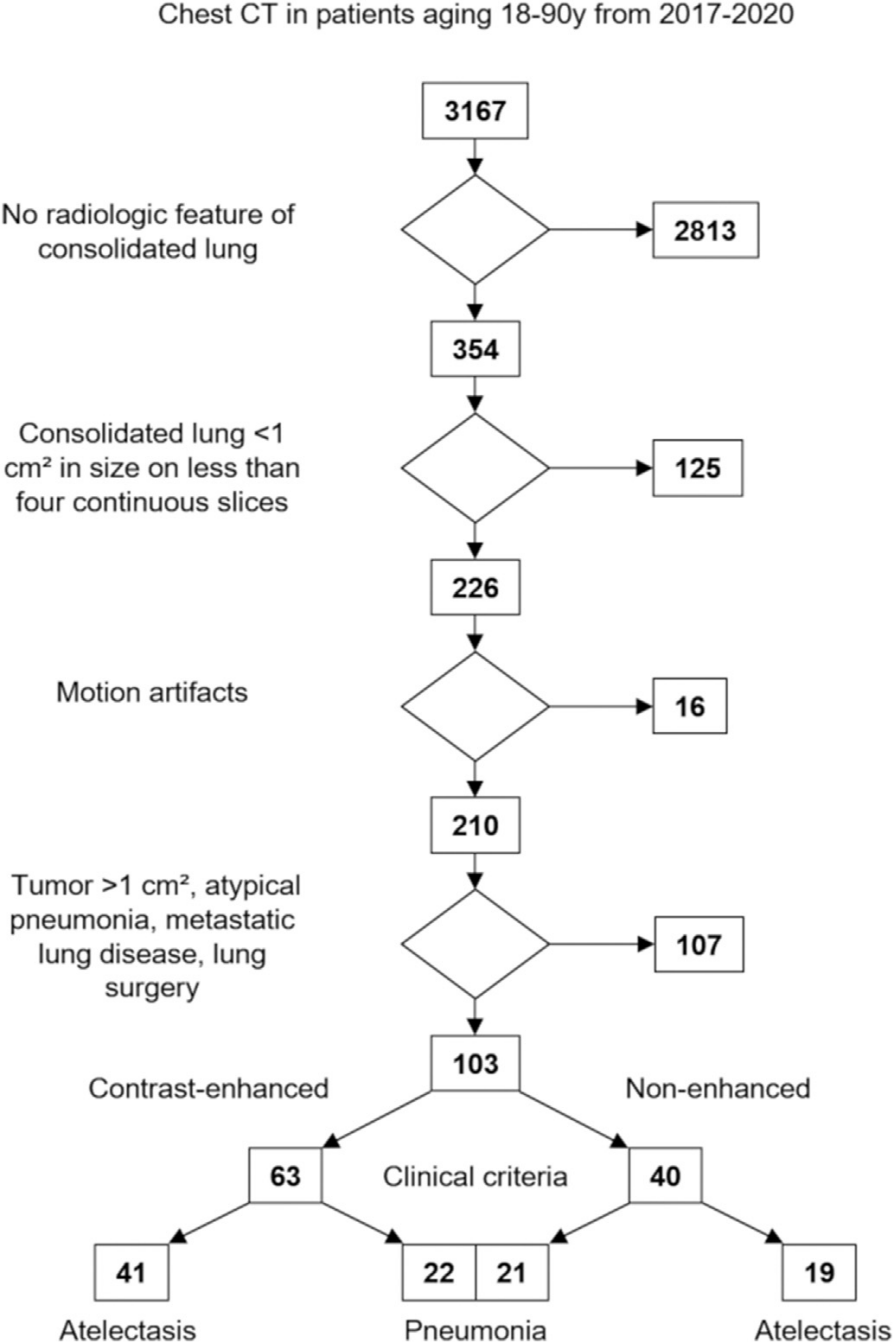
Flowchart for patient recruitment. Database research encompassing the years 2017–2020 identified 3167 patients who underwent chest CT. Two hundred twenty-six patients had consolidated lung >1 cm^2^ in size on four continuous slices as an imaging feature. Out of these, 123 patients were excluded due to motion artifacts, tumor >1 cm^2^, radiologic features of atypical pneumonia, metastatic lung disease, or previous lung surgery. The remaining 103 patients were split up in contrast-enhanced and non-contrast CT. Finally, the patients were classified as atelectasis or pneumonia based on clinical criteria.

CT acquisitions were classified as atelectasis or pneumonia based on the presence of a non-radiologic clinical scoring system adapted from Edwards et al. [6]. Clinical criteria were (1) hypoxia, tachypnea, grunting, chest in drawing and/or crackles on auscultation, (2) blood testing (C-reactive protein (CRP) > 5 mg/l or white blood cell count (WBC) >4–10/ml), (3) antibiotic treatment (Abx.) for pneumonia (aminopenicillin and/or after admission in the hospital with a second- or third-generation cephalosporin) or (4) documentation of pneumonia as a discharge diagnosis or clinical suspicion of pneumonia as an indication for chest CT. One point was given for the presence of each criterion. Each case was classified as atelectasis if one or less, and as pneumonia if three or more criteria were met. If two criteria were met, patients with an apparent non-pulmonary infection were also classified as atelectasis, and patients with cough and no other apparent non-pulmonary infections were classified as pneumonia (Figure 1).

### 2.2 CT acquisition

All SDCT examinations were performed using a 128-slice dual-layer CT system (IQon; Philips GmbH, Hamburg, Germany). All patients were acquired in supine position during an inspirational breath-hold in the craniocaudal direction. The following acquisition parameters were used: collimation 2 × 64 × 0.625 mm; rotation time 0.33–0.75 s; pitch 0.798–1.014; tube current 120 kVp, dose modulation type: DoseRight 3D-DOM with an Dose Right Index (DRI) of 17 (Philips GmbH, Hamburg, Germany). All images were reconstructed in axial orientation using an image matrix of 512 × 512 with a slice thickness of 1-1,5 mm and an increment of 0.75–3 mm using a dedicated spectral reconstruction algorithm (Spectral, Philips GmbH, Hamburg, Germany) and a fixed kernel (B; Philips GmbH, Hamburg, Germany). Conventional 120 kVp (CON_120kVp_), iodine concentration (C_iodine_), and effective atomic number (Z_eff_) images were reconstructed (Table 1).

**Table 1:** SDCT acquisition parameters.

Protocol	Collimation (mm)	Image matrix	Pitch	Gantry rotation time (s)	Acquisition time (s)	Tube current (kVp)	Tube current-time product (mAs)	Absolute Min (mAs)	Absolute Max (mAs)
Native chest	2 × 64×0.625	512 × 512	1.014	0.75	7.8	120	47	35	230
Venous chest	2 × 64×0.625	512 × 512	0.984	0.33	3.5	120	93	40	300
Venous body	2 × 64×0.625	512 × 512	0.798	0.5	9.6	120	74	65	300

In all patients who underwent contrast-enhanced CT, the contrast media (350 mg Iohexol/ml; AccupaqueTM 350; GE Healthcare GmbH, Solingen, Germany) was injected via an antecubital vein or a central venous catheter using a power injector. Venous phase imaging was triggered by bolus tracking at the level of the truncus pulmonalis with a threshold of 150 HU and a post-threshold-delay of 35 s. Single bolus contrast-injection was performed for venous chest acquisitions administering 50 ml contrast media followed by a 60 ml saline solution chaser bolus at a 4 ml/s flow rate. Venous body acquisitions used a biphasic contrast-injection protocol consisting of two contrast boli, the first with 50 and the second with 40 ml contrast agent, both followed by a saline solution chaser bolus of 15 and 30 ml, respectively. The interval between both boluses was 30 s, and the flow rate was 3 ml/s. All protocols were slightly adjusted to the patients' body weight.

### 2.3 Image analysis

Two readers with two and seven years of experience in thoracic imaging analyzed the images, using a picture archiving and communication system (PACS) workstation (Centricity, Version 7.0; General Electrics, New York, USA) and a dedicated post-processing software provided by the SDCT manufacturer (IntelliSpace Portal 10; Philips GmbH, Hamburg, Germany). Images were read in a non-randomized fashion, and the regions of interest (ROIs) were placed in consensus. On conventional images, two standardized oval ROIs of 1 cm^2^ and one maximum-sized ROI were placed each in the consolidated lung (pneumonia or atelectasis), the normal lung, and pleural effusion (PE), excluding bronchi and third-order or larger pulmonary vessels. Normal lung regions were chosen by absence of consolidation or ground glass attenuation at the hilum level and at least 1 cm away from the pleura. Singular vessel ROIs were placed in the ascending aorta (AA) and the right pulmonary artery (RPA) at the right proximal pulmonary artery level. ROIs placed on conventional images were automatically transferred to the corresponding position on the iodine concentration (C_iodine_) and the effective atomic number (Z_eff_) images. For each ROI, absolute attenuation values in Hounsfield units (HU), iodine concentration (mg/ml) and effective atomic number as well as respective standard deviations (SD) were recorded. Measurements of two ROIs were averaged. According to the definition of van Engen *et al.*, the contrast-to-noise ratio (CNR) between two tissues (1 and 2) was defined as $\;CNR=|{\text{S}}_{1}-{\text{S}}_{2}|/\sqrt{0.5(\sigma_{1}^{2}\;+\;\sigma_{2}^{2}})$ with S being the averaged HU on CON_120kVp_, iodine concentration (C_iodine_) or effective atomic number (Z_eff_) in two homogeneous ROI, and σ being the standard deviation in the same ROIs [16].

### 2.4 Statistical analysis

All data were recorded in a dedicated spreadsheet (Excel, Microsoft Corp., Redmond, USA), and analyses were performed with SigmaPlot (Systat Software GmbH, Erkrath, Germany) and SPSS (IBM SPSS Statistics 25, New York; USA). All data are given as mean ± standard deviation (SD). Quantitative imaging parameters results were tested for significant differences with the Mann-Whitney Rank Sum Test for non-paired measurements. An additional analysis using the Bonferroni-Holm method for multiple testing was performed, which did not change the number of significant results. Receiver operating characteristic (ROC) analysis was used to evaluate the performance of conventional (CON_120kVp_), iodine concentration (C_iodine_), and effective atomic number (Z_eff_) images in discriminating between pneumonia and atelectasis. The overall performance was summarized using the area under the curve (AUC). Thresholds were chosen by maximizing the Youden index (J = sensitivity + specificity −1), which treats sensitivity and specificity as equally important and is not weighted by the pre-test probability [17]. Statistical significance was defined as p ≤ 0.05. CNRs of all three quantitative parameters were compared using one-way analysis of variances (ANOVA) for repeated measures, and post-hoc tests with Bonferroni's correction or Dunn's method as appropriate in case of multiple comparisons. In addition, for each data set a feature vector was composed out of the HU, C_iodine_ and Z_eff_ values of the pneumonia, aorta and atelectasis region. Subsequently, principle component analysis (PCA) implemented in the freely available analysis software PAleontological STatistics (PAST) was performed in a 9 dimensional feature space separately for the data sets with and without contrast enhancement [18, 19].

## 3 Results

### 3.1 Patient cohort

In total, 103 patients aged 62.2 ± 16.6 years (range: 19–88 years) could be recruited. Sixty-three patients, 22 clinically diagnosed with pneumonia and 41 with atelectasis, underwent contrast-enhanced SDCT, and 40 patients, 21 clinically diagnosed with pneumonia, and 19 with atelectasis, underwent non-enhanced chest SDCT. There were no significant differences in age (p = 0.574, p = 0.818) or BMI (p = 0.833; p = 0.483), when comparing both groups (Table 2). In both atelectasis groups, 55 % of cases had less than two clinical criteria, which allowed an exact classification. 42 % of cases fulfilled two clinical criteria, but since all of them had an apparent non-pulmonary infection, they were also classified as atelectasis. In both pneumonia groups, 70 % of cases had more than two clinical criteria and were therefore classified as pneumonia. 30 % of cases fulfilled only two clinical criteria, but most of them had a cough, and no other apparent non-pulmonary infection was found; therefore, they were classified as pneumonia (Table 2).

**Table 2:** Patient demographics and diagnostic criteria for patients with contrast-enhanced and non-enhanced chest CT.

Patient demographics	Contrast-enhanced			Non-enhanced		
	Atelectasis	Pneumonia	p	Atelectasis	Pneumonia	p
N	41	22	-	19	21	-
Sex (m/f)	20/21	12/10	-	9/10	13/8	-
Age (y)	64.4 ± 15.1	66.8 ± 14.3	0.574	55.5 ± 20.2	59.3 ± 15.5	0.818
BMI (kg/cm^2^)	27.7 ± 5.4	28.9 ± 8.2	0.833	24.5 ± 4.3	28.4 ± 4.1	0.483
**Diagnostic criteria**						
Fever and/or cough	2 (5 %)	11 (50 %)	-	7 (37 %)	17 (81 %)	-
Leucocytosis and/or CRP	20 (49 %)	11 (50 %)	-	3 (16 %)	6 (29 %)	-
Abx. treatment	25 (61 %)	21 (95 %)	-	11 (58 %)	18 (86 %)	-
Clinical diagnosis of pneumonia	2 (5 %)	22 (100 %)	-	1 (5 %)	21 (100 %)	-
Nonpulmonary septic foci	27 (66 %)	4 (18 %)	-	13 (68 %)	3 (14 %)	-
**Total no. criteria present**						
0 of 4	12 (29 %)	0 (0 %)	-	7 (37 %)	0 (0 %)	-
1 of 4	11 (27 %)	0 (0 %)	-	3 (16 %)	0 (0 %)	-
2 of 4	18 (39 %)	9 (41 %)	-	9 (47 %)	4 (19 %)	-
3 of 4	0 (0 %)	5 (23 %)	-	0 (0 %)	11 (52 %)	-
4 of 4	0 (0 %)	8 (36 %)	-	0 (0 %)	6 (29 %)	-

Patient characteristics given as median and standard deviation. BMI = body mass index. Distribution of diagnostic criteria with the final clinical diagnosis of atelectasis or pneumonia. Abx indicates antibiotics.

### 3.2 Influence of ROI sizes and contrast-phase on quantitative CT parameters

Quantitative CT parameters were compared between standardized and maximum-sized ROI, and no significant differences were found in the atelectasis or the pneumonia group (p = 0.953, p = 0.683) (Table 3).

**Table 3:** Influence of ROI sizes on the quantitative CT parameters.

	Atelectasis			Pneumonia		
	standardized	maximum-sized	p	standardized	maximum-sized	p
**Region of interest (mm**^**2**^**)**						
Normal lung	10 ± 0	46 ± 12	<0.001	10 ± 0	55.51 ± 16.03	<0.001
Consolidated lung	10 ± 0	19 ± 7	<0.001	10 ± 0	22.01 ± 8.80	<0.001
Pleural effusion	10 ± 0	32 ± 18	<0.001	10 ± 0	15.87 ± 8.74	<0.001
**CT numbers measured on CON**_**120kVp**_**(HU)**						
Normal lung	-814 ± 57	-797 ± 47	0.055	-810 ± 65	-816.98 ± 52.30	0.953
Consolidated lung	105 ± 21	102 ± 21	0.683	60 ± 13	62.23 ± 13.34	0.869
Pleural effusion	9 ± 8	10 ± 11	0.514	6 ± 11	11.94 ± 14.11	0.536
**Iodine concentration measured on C**_**iodine**_**(mg/ml)**						
Normal lung	0.71 ± 0.39	0.68 ± 0.20	0.468	0.67 ± 0.39	0.70 ± 0.23	0.230
Consolidated lung	2.65 ± 0.94	2.59 ± 0.92	0.809	1.28 ± 0.39	1.28 ± 0.50	0.824
Pleural effusion	0.06 ± 0.12	0.09 ± 0.17	0.705	0.09 ± 0.08	0.10 ± 0.12	0.569
**Effective atomic number measured on Z**_**eff**_						
Normal lung	9.37 ± 0.57	9.52 ± 0.66	0.356	9.36 ± 0.65	9.42 ± 0.60	0.681
Consolidated lung	8.63 ± 0.39	8.62 ± 0.39	0.937	8.03 ± 0.20	8.03 ± 0.26	0.742
Pleural effusion	7.19 ± 0.17	7.22 ± 0.21	0.350	7.24 ± 0.16	7.26 ± 0.17	0.878

Standardized ROI (2 × 1mm^2^) were compared with ROIs of maximum size. Mean ± standard deviation for CT numbers on conventional (CON_120kVp_) images as well as iodine concentration (C_iodine_) and effective atomic number (Z_eff_) on spectral images in the atelectasis and the pneumonia group. Pleural effusion was present in n = 13 patients in the atelectasis, and never in the pneumonia group.

In 63 patients contrast medium was applied intravenously and no significant differences between the atelectasis and pneumonia group were found for volume (ml), duration (sec) and flow rate (ml/s) (p = 0.667, p = 0.527, p = 0.088). ROIs were used to determine the average attenuation in the descending aorta (DA) and the right pulmonary artery (RPA), also showing no significant differences between both groups (p = 0.846, p = 0.941, p = 0.915) (Table 4).

**Table 4:** Contrast administration protocol and measurements in vessel ROIs on contrast-enhanced chest CT.

	Atelectasis	Pneumonia	p
**Contrast administration protocol**			
Volume (ml)	86.68 ± 19.19	80.95 ± 22.36	0.667
Duration (s)	28.04 ± 5.85	24.92 ± 5.70	0.527
Flow rate (ml/s)	3.10 ± 0.42	3.22 ± 0.43	0.088
**CT numbers in vessel ROIs (HU)**			
Descending aorta	193 ± 82	197 ± 83	0.846
Right pulmonary artery	179 ± 83	153 ± 21	0.941
Average of DA and RPA	187 ± 83	178 ± 68	0.915

Contrast media volume, application duration, and flow rate are given for the atelectasis and the pneumonia group. Contrast administration data was missing for 12 patients. The CT number values in Hounsfield units on conventional images are given for the descending aorta (DA) and the right pulmonary artery (RPA). Both measurements were also averaged.

### 3.3 Contrast media is needed for quantitative discrimination between atelectasis and pneumonia

**Figure 2:**
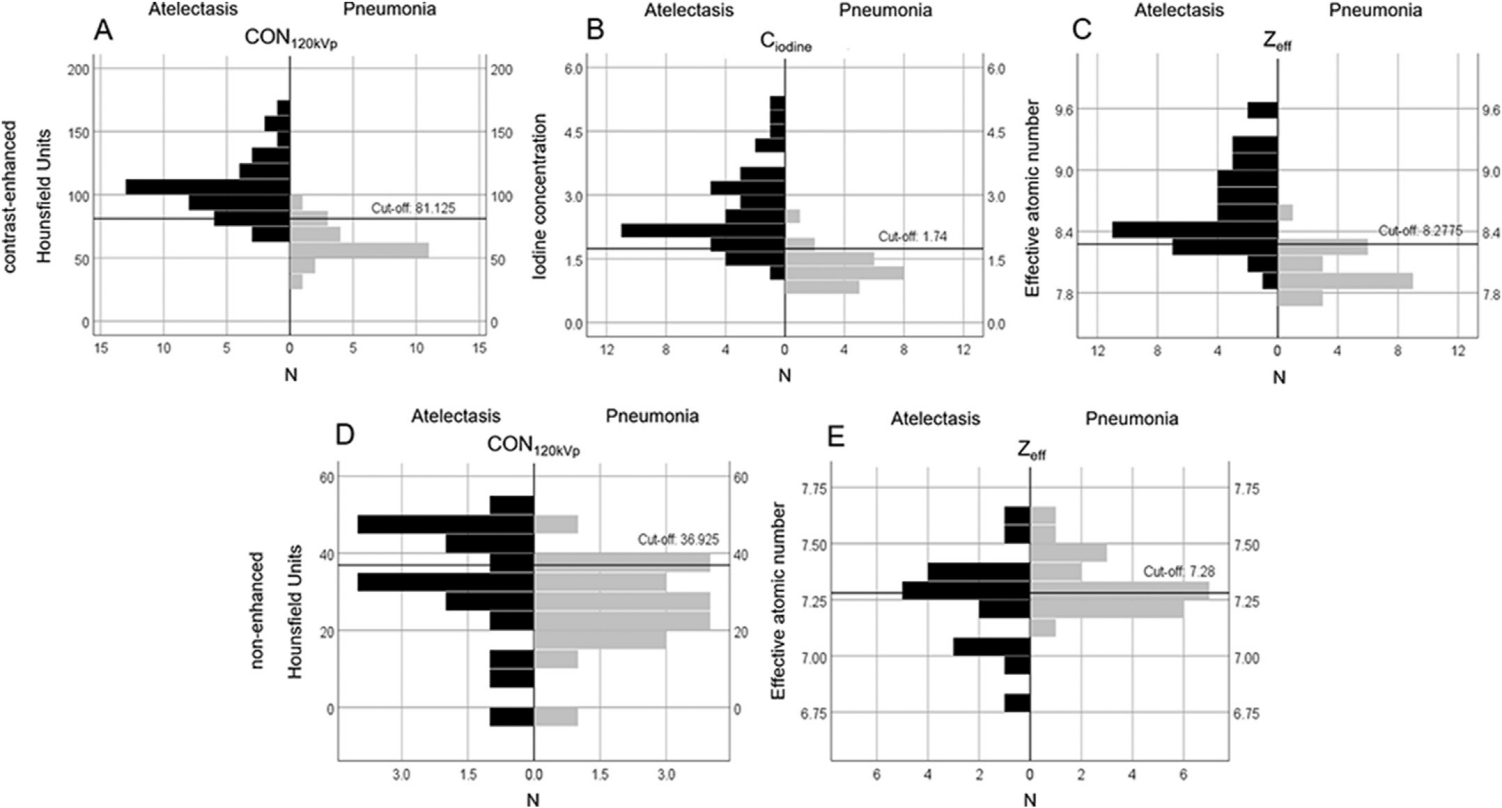
Boxplots for quantitative CT measurements. Values are given for the regions of interest on (A, D) conventional (CON_120kVp_), (B) iodine concentration (C_iodine_), and (C, F) effective atomic number (Z_eff_) images for atelectasis and the pneumonia for all patients (N). The cut-off values were calculated using the Youden-Index.

On non-enhanced SDCT, no significant differences were found between the atelectasis and the pneumonia group, neither on CON_120kVp_ (p = 0.054) nor on Z_eff_ images (p = 0.563) (Figure 2 and Table 5). The AUCs for non-enhanced images were expectedly small, with the largest AUC 0.68 (0.50–0.86) achieved on CON_120kVp_ images with a sensitivity of 56 % and specificity of 52 %. Z_eff_ had an AUC of 0.57 (0.26–0.64) with a slightly higher sensitivity of 61 % and the same specificity.

**Table 5:** Quantitative parameters on non-enhanced chest SDCT.

	Atelectasis	Pneumonia	p
**CT numbers measured on CON**_**120kVp**_**(HU)**			
Normal lung	-833 ± 58	-835 ± 56	0.789
Consolidated lung	37 ± 15	26 ± 10	0.054
Pleural effusion	1 ± 9	-	-
**Effective atomic number measured on Z**_**eff**_			
Normal lung	7.91 ± 0.49	7.65 ± 0.44	0.076
Consolidated lung	7.24 ± 0.20	7.31 ± 0.12	0.563
Pleural effusion	7.10 ± 0.14	-	-

Mean ± standard deviation for ROIs on conventional (CON_120kVp_) and effective atomic number (Z_eff_) images are given for the atelectasis and the pneumonia group. Pleural effusion was present in n = 13 patients in the atelectasis, and never in the pneumonia group.

### 3.4 Quantitative parameters can discriminate atelectasis from pneumonia with contrast media

**Figure 3:**
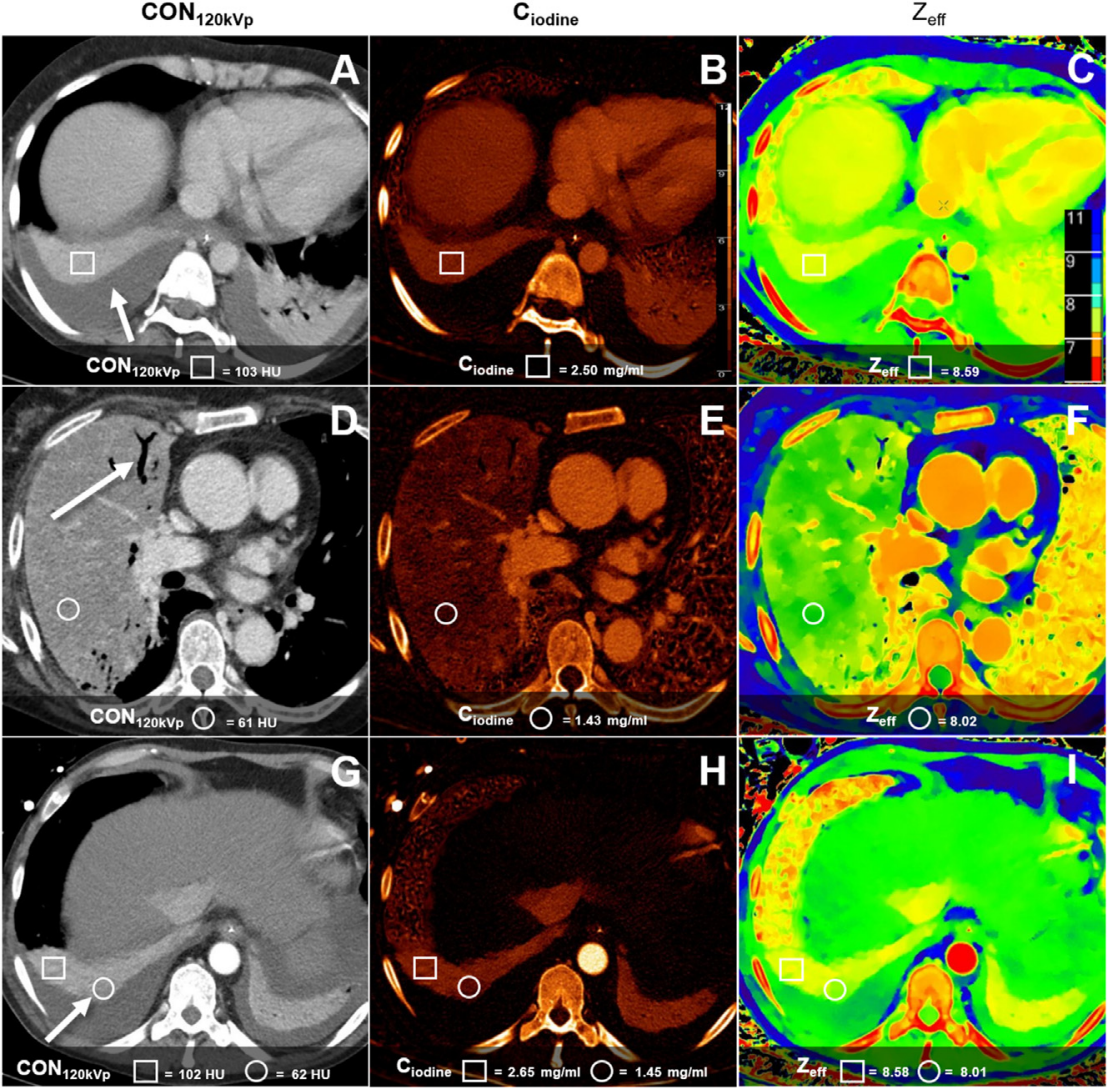
Conventional, iodine concentration, and effective atomic number images of contrast-enhanced chest CT in three different patients. (A, B, C) Bilateral pleura effusion within adjacent homogenous atelectasis (white arrow) in a 45-year-old woman. (D, E, F) Right-sided lobar pneumonia with a positive air bronchogram a 51-year-old woman. (G, H, I) Bilateral pleural effusion with adjacent atelectasis in a 45-year-old man. On the right side is a focal area with hypoperfusion within the atelectasis (white arrow), which is suspicious for a superinfection of the atelectasis. Quantitative CT parameters are shown for atelectasis (square) and pneumonia (circle).

On contrast-enhanced SDCT, CT numbers of the consolidated lung were significantly higher in the atelectasis group than in the pneumonia group for CON_120kVp_, (p < 0.001). Correspondingly, C_iodine_, and Z_eff_ were also significantly higher in the atelectasis group (p < 0.001). We found no significant differences for normal lung or pleural effusion measurements between both groups (Figure 2, Figure 3, and Table 6).

**Table 6:** Quantitative parameters on contrast-enhanced chest SDCT.

	Atelectasis	Pneumonia	p
**CT numbers measured on CON**_**120kVp**_**(HU)**			
Normal Lung	-814 ± 57	-810 ± 65	0.971
Consolidated lung	105 ± 21	60 ± 13	<0.001
Pleural effusion	9 ± 8	6 ± 11	0.723
**Iodine concentration measured on C**_**iodine**_**(mg/ml)**			
Normal lung	0.71 ± 0.39	0.67 ± 0.39	0.498
Consolidated lung	2.65 ± 0.94	1.28 ± 0.39	<0.001
Pleural effusion	0.06 ± 0.12	0.09 ± 0.08	0.077
**Effective atomic number measured on Z**_**eff**_			
Normal lung	9.37 ± 0.57	9.36 ± 0.65	0.943
Consolidated lung	8.63 ± 0.39	8.03 ± 0.20	<0.001
Pleural effusion	7.19 ± 0.17	7.24 ± 0.16	0.180

Mean ± standard deviation for ROIs on conventional (CON_120kVp_), iodine concentration (C_iodine_), and effective atomic number (Z_eff_) images are given for the atelectasis and the pneumonia group. Pleural effusion was present in n = 29 patients in the atelectasis, and in n = 17 patients in the pneumonia group.

### 3.5 Best contrast-to-noise ratio was achieved on conventional images

On CON_120kVp_ images, significantly higher CNRs were achieved between normal lung and atelectasis or pneumonia compared to the C_iodine_ and Z_eff_ images (p < 0.001). On the Z_eff_ images, the highest CNRs were found between the aorta, pleural effusion, and atelectasis or pneumonia (p < 0.001) (Table 7).

**Table 7:** Contrast-to-noise ratios for consolidated lung.

	CON_120kVp_	C_iodine_	Z_eff_	p
**Atelectasis**				
vs. aorta	4.32	6.91	24.83	<0.001
vs. normal lung	30.63	3.54	4.22	<0.001
vs. pleural effusion	5.21	5.01	23.37	<0.001
**Pneumonia**				
vs. aorta	7.14	9.79	37.52	<0.001
vs. normal lung	27.69	1.27	7.63	<0.001
vs. pleural effusion	2.98	2.63	7.30	<0.001

Contrast-to-noise ratios between atelectasis or pneumonia and the aorta, normal lung and pleural effusion was calculated using conventional (CON_120kVp_), iodine concentration (C_iodine_), and effective atomic number (Z_eff_) images.

### 3.6 All quantitative parameters showed comparable diagnostic properties

**Figure 4:**
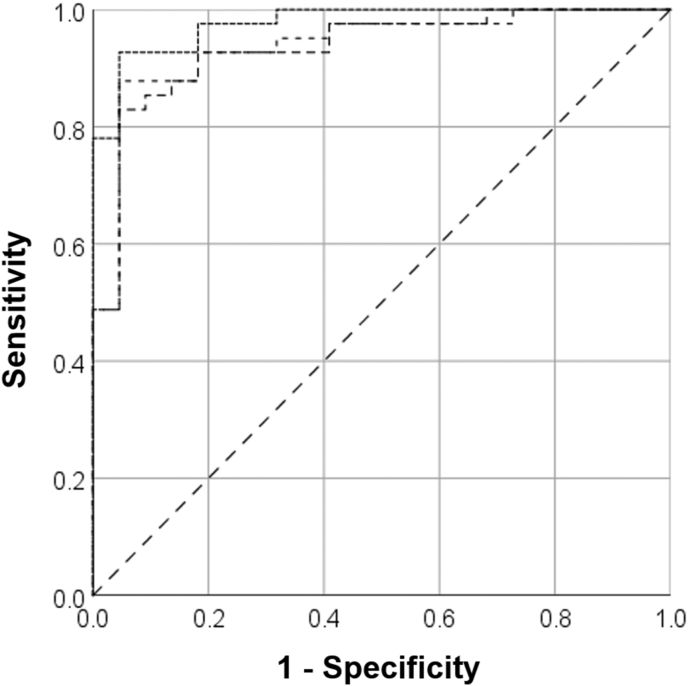
The area under the curve for diagnosis of pneumonia for quantitative SDCT parameters. Sensitivity and specificity for pneumonia on contrast-enhanced (A) and non-enhanced (B) SDCT for conventional attenuation (CON_120kVp_), iodine concentration (C_iodine_), and effective atomic number (Z_eff_) images.

On contrast-enhanced SDCT, the AUC to differentiate atelectasis from pneumonia on CON_120kVp_ images was 0.98 (CI 0.94–0.99), whereas it was slightly lower for C_iodine_ and Z_eff_ images, with 0.94 (CI 0.87–0.99) and 0.93 (CI 0.87–0.99), respectively. On CON_120kVp_ images, a threshold of 81 HU achieved a sensitivity of 93 % and specificity of 95 % for identifying pneumonia. C_iodine_, and Z_eff_ images reached comparable sensitivities of 95 % when using a threshold of 1.74 mg/ml and 8.27, but with somewhat lower specificities of 85 % and 83 %, respectively (p < 0.001) (Figure 4 and Table 8).

**Table 8:** Sensitivity and specificity of quantitative SDCT parameters for discriminating atelectasis from pneumonia.

AUC (95% Cl)	Threshold	Sensitivity (%)	Specificity (%)	p
**CT numbers measured on CON**_**120kVp**_**(HU)**				
0.98 (0.94–0.99)	81	93	95	<0.001
**Iodine concentration measured on C**_**iodine**_**(mg/ml)**				
0.94 (0.87–0.99)	1.74	85	95	<0.001
**Effective atomic number measured on Z**_**eff**_				
0.93 (0.87–0.99)	8.27	83	95	<0.001

Sensitivity and specificity as well as area under the curve (AUC) for detecting pneumonia on contrast-enhanced chest SDCT. Thresholds were chosen for conventional (CON_120kVp_), iodine concentration (C_iodine_), and effective atomic number (Z_eff_) images by maximizing the Youden index.

### 3.7 Diagnosis can be based CT on number measurements

**Figure 5:**
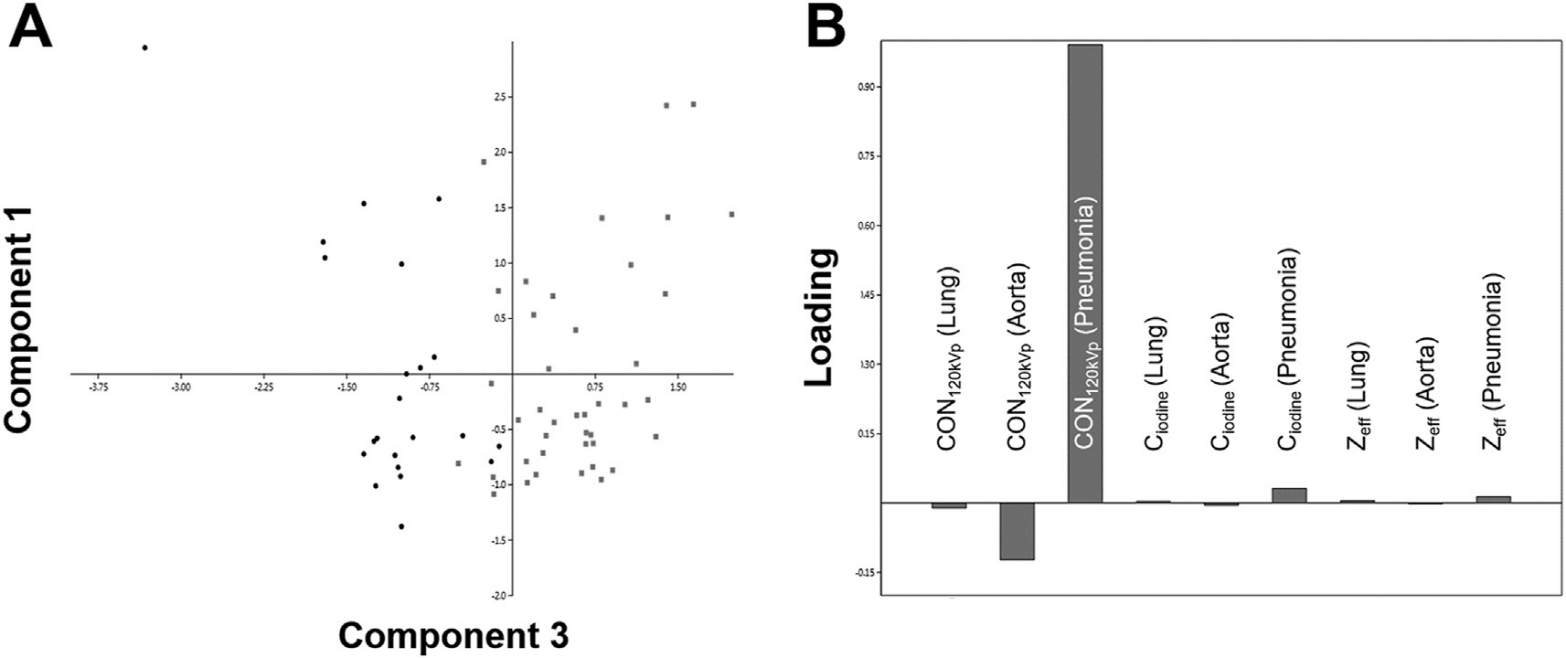
The principal component analysis (PCA). (A) PCA biplot shows both the component scores of pneumonia (dots) and atelectasis (squares). (B) Loadings for conventional (CON_120kVp_, Hounsfield Units [HU]), iodine concentration (C_iodine_, [mg/ml]), and effective atomic number (Z_eff_) for lung, the aorta and pneumonia refer to the Component 3 axis. The graph shows, that the differentiation between atelectasis and pneumonia is almost entirely based on the CT number in HU of pneumonia and to a lesser extent of the CT number in HU of the aorta. The other parameters were irrelevant.

The principal component analysis (PCA) showed the highest variance on the CT number axis, whereas the other parameters only showed little variance, implying that the criterion CT number alone is enough to decide between atelectasis and pneumonia (Figure 5).

## 4 Discussion

This study investigated the value of SDCT for quantitative differentiation between atelectasis and pneumonia on contrast-enhanced chest CT. We showed that the quantitative parameters C_iodine_ and Z_eff_ could distinguish atelectasis and pneumonia but without added value compared to CT number measurements on conventional images. As expected, in a negative control group of 40 non-enhanced CT, none of the quantitative parameters could differentiate atelectasis and pneumonia.

CT imaging plays an important role since radiologic findings are considered in the diagnosis. The consolidated lung is a frequent finding in chest CT, in which the air within the affected airspaces is replaced, increasing the pulmonary attenuation and obscuring the margins of adjacent airways and vessels [4]. The radiologist needs to differentiate infectious consolidations caused by pneumonia from non-infectious consolidations caused by atelectasis. Radiological signs suggesting pulmonary infection are the air bronchogram, a volume increase, vessel or airway crowding, adjacent ground glass, or tree-in-bud opacities [4, 5]. However, although radiologic signs may help to assign consolidations to an infectious or non-infectious cause, they remain qualitative non-obligatory observations. Therefore, the diagnosis might be unsure, for example, in patients with underlying chronic disease such as heart failure and pleural effusion, who frequently have basal atelectasis that cannot be reliably distinguished from parenchymal infection [20]. In these cases, quantitative CT parameters would be desirable to facilitate a more confident diagnosis.

Atelectasis and pneumonia differ in tissue density and contrast enhancement which can be detected by quantitative CT parameters. In pneumonia, the volume of the affected lung tissue increases since the alveolar airspaces are filled with fluid or cells, whereas in atelectasis, the lung volume is reduced by compression, absorption of alveolar air, or impaired pulmonary surfactant production or function [21]. Therefore, the lung tissue density per voxel is higher in atelectasis. The contrast enhancement will be influenced by the number of capillaries per voxel and the complex interaction of pathophysiological mechanisms regulating local perfusion. Atelectasis and pneumonia are causing regional alveolar hypoxia leading to hypoxic pulmonary vasoconstriction and reducing pulmonary perfusion regionally, while active inflammation in pneumonia may increase perfusion [22]. However, the volume effect seems to have a more substantial influence since a higher contrast enhancement can be overserved in atelectasis, represented by higher CT numbers and higher SPCT parameters values.

The CT number is a relative quantitative measurement of x-ray density and the most frequently used quantitative CT parameter. On non-enhanced images, the difference in tissue density alone is usually not significant enough to make a sure distinction between atelectasis and pneumonia, which we confirmed by measuring a non-significant difference of 11°HU (p = 0.054). Edwards et al. reported a threshold of 85 HU with a sensitivity of 90 % and a specificity of 92 % for pneumonia by using their contrast-enhanced pulmonary CT angiogram protocol [6]. In our study, the difference between atelectasis and pneumonia was also significant on CON_120kVp_, achieving a sensitivity of 93 % and specificity of 95 %. However, in our study, the optimal threshold was slightly lower with 81 HU. Nonetheless, we believe that both thresholds are comparable. Edwards *et al.* reported a median value of 119 HU for atelectasis and 62 HU for pneumonia, whereas we determined means of 105 HU and 60 HU, respectively. They also reported higher averaged CT numbers of 252 HU and 278 HU than ours, with 187 HU and 178 HU for the ROIs placed in the ascending aorta and the right pulmonary artery. The higher HU values reported by Edwards *et al.* imply higher concentrations of contrast material and can be explained by their triggered arterial phase CT angiogram protocol with a minimal acquisition delay of around 8–10 s [23]. Our study's venous chest and body protocols had longer acquisition delays of 30–45 s depending on the patient's cardiac output. Furthermore, they administered their contrast material with a higher flow rate of 4–5 ml/s than ours of 1.98–3.92 ml/s. In summary, our data showed that pneumonia could be diagnosed with high sensitivity and specificity on CON_120kVp_, which is following the existing literature.

SDCT can provide additional material-nonspecific and material-specific energy-dependent information like iodine concentration (C_iodine_) and the effective atomic number (Z_eff_). We assumed that these parameters might offer a better differentiation of atelectasis and pneumonia by depicting microvessel density and blood supply. In this context, significant correlations between iodine uptake and perfusion parameters derived from DECT and first-pass dual-input perfusion computed tomography (DIPCT) have been reported [24]. Furthermore, SDCT parameters were already used to differentiate lung cancer from inflammatory masses and to detect pulmonary embolism and pleural contrast uptake [13, 14, 15]. As expected, C_iodine_ and Z_eff_ showed significantly higher values in the atelectasis group (p < 0.001), while both parameters showed comparable sensitivities of 95 %, but overall lower specificities of 85 % and 83 %. Therefore, both parameters had no added diagnostic value compared to CT number measurements on conventional images. This conclusion was also strengthened by principal component analysis, which showed that the differentiation between atelectasis and pneumonia could be solely based on CT numbers measurements.

We also investigated whether spectral images have better contrast-to-noise-ratios. The ratio between the contrast of two adjacent structures and the noise level are two major criteria to assess the ability to separate different structures. We calculated the CNR values between the aorta, normal lung, and pleural effusion vs. atelectasis or pneumonia. CON_120kVp_ images had significantly higher CNRs between consolidated and normal lung than the corresponding C_iodine_ and Z_eff_ images (p < 0.001). On spectral images, the noise increases dramatically if the spectral resolution is low [25]. Therefore, the lower CNRs are most likely caused by a higher noise level even though contrast may be enhanced. On Z_eff_ images, significantly higher CNRs were found between the aorta or pleural effusion and atelectasis or pneumonia (p < 0.001). The reason for this is because blood and pleural effusion consist mostly of inorganic materials, which is reflected by Z_eff_. In summary, C_iodine_ and Z_eff_ had no benefit compared to CON_120kVp_ images on non-enhanced chest SDCT with regard to CNR.

There are some technical limitations to our study. First, a validated gold standard is missing, as no histological correlation was performed. Bronchoalveolar lavage or lung biopsy are the reference methods for diagnosing pneumonia but are seldom performed and were not available in a retrospective setting. The clinical criteria we used to assign patients to the pneumonia group have been used slightly modified in other studies [6, 26, 27]. Unfortunately, there were many multimorbid patients in our patient cohort who often had several extrapulmonary infections. A considerable part of the patients had a clinical score of two, which did not clearly assign them to the atelectasis or the pneumonia group. In these cases, individual decisions were made, which was challenging due to the partially overlapping clinical symptoms of atelectasis and pneumonia [28]. Nevertheless, Edwards *et al.* reported comparable results for CT number measurements on CON_120kVp_, implying that our clinical assignment was adequate enough to allow the assessment of C_iodine_ and Z_eff_ images. Furthermore, we ignored the intra- and inter-individual differences in iodine distribution, which can be seen as another limitation. In the literature, significant differences in iodine concentrations were reported between sexes and age in different parenchymal organs, influencing the obtained quantitative iodine concentration and the applied iodine thresholds [29]. However, even though comparable effects can be expected in the lungs, we argue that our calculated thresholds are still valid since they are based on averaged values, by which the impact of intra- and inter-individual differences are reduced.

## 5 Conclusion

We showed that the quantitative parameters C_iodine_ and Z_eff_ could distinguish atelectasis and pneumonia in contrast-enhanced SDCT but without added diagnostic value compared to CT number measurements on conventional images. Thus, in every day routine CT contrast material application can add diagnostic value based on quantitative measurements in cases where radiological and clinical diagnosis both are equivocal.

## Declarations

### Author contribution statement

Philip Konietzke and Mark Oliver Wielpütz: Conceived and designed the experiments; Performed the experiments; Analyzed and interpreted the data; Contributed reagents, materials, analysis tools or data; Wrote the paper.

Hauke Helmke Steentoft: Performed the experiments; Analyzed and interpreted the data; Contributed reagents, materials, analysis tools or data; Wrote the paper.

Willi Linus Wagner: Contributed reagents, materials, analysis tools or data; Wrote the paper.

Jonas Albers and Christian Dullin: Analyzed and interpreted the data.

Stephan Skornitzke: Analyzed and interpreted the data; Contributed reagents, materials, analysis tools or data; Wrote the paper.

Wolfram Stiller and Tim Frederik Weber: Conceived and designed the experiments; Analyzed and interpreted the data; Contributed reagents, materials, analysis tools or data; Wrote the paper.

Hans-Ulrich Kauczor: Conceived and designed the experiments; Contributed reagents, materials, analysis tools or data; Wrote the paper.

### Funding statement

This work was supported by 10.13039/501100002347Federal Ministry of Education and Research (82DZL004A, 82DZL004A2).

### Data availability statement

Data included in article/supplementary material/referenced in article.

### Declaration of interests statement

The authors declare the following conflict of interests: Stephan Skornitzke has ownership interests in investment funds containing stock of healthcare companies.

### Additional information

No additional information is available for this paper.
